# Effect of Pd Precursor Salts on the Chemical State, Particle Size, and Performance of Activated Carbon-Supported Pd Catalysts for the Selective Hydrogenation of Palm Biodiesel

**DOI:** 10.3390/ijms22031256

**Published:** 2021-01-27

**Authors:** Parncheewa Udomsap, Apiluck Eiad-Ua, Shih-Yuan Chen, Takehisa Mochizuki, Nuwong Chollacoop, Yuji Yoshimura, Masayasu Nishi, Hiroyuki Tateno, Hideyuki Takagi

**Affiliations:** 1 College of Nanotechnology, King Mongkut’s Institute of Technology Ladkrabang, Chalongkrung Rd., Ladkrabang, Bangkok 10520, Thailand; parncheewa.udo@entec.or.th; 2 Energy Catalyst Technology Group, Energy Process Research Institute (EPRI), National Institute of Advanced Industrial Science and Technology (AIST), 16-1 Onogawa, Tsukuba, Ibaraki 305-8559, Japan; m.nishi@aist.go.jp (M.N.); tateno-hiroyuki@aist.go.jp (H.T.); hide-takagi@aist.go.jp (H.T.); 3 Energy Innovation Research Group, National Energy Technology Center (ENTEC), 114 Thailand Science Park, Phaholyothin Rd., Klong 1, Klong Luang, Pathumthani 12120, Thailand; nuwong.cho@entec.or.th (N.C.); y.yoshimura@opal.ocn.ne.jp (Y.Y.); 4 Hydrogen Production and Storage Team, Global Zero Emission Research Center (GZR), National Institute of Advanced Industrial Science and Technology (AIST), 16-1 Onogawa, Tsukuba, Ibaraki 305-8559, Japan

**Keywords:** Pd catalyst, activated carbon, particle size effect, selective hydrogenation, high-quality biodiesel

## Abstract

To improve the oxidative stability of biodiesel fuel (BDF), the polyunsaturated fatty acid methyl esters (poly-FAME) presented in commercial palm oil-derived biodiesel fuel (palm-BDF) were selectively hydrogenated to monounsaturated fatty acid methyl esters (mono-FAME) under a mild condition (80 °C, 0.5 MPa) using activated carbon (AC)-supported Pd catalysts with a Pd loading of 1 wt.%. The partially hydrotreated palm-BDF (denoted as H-FAME) which has low poly-FAME components is a new type of BDF with enhanced quality for use in high blends. In this study, we reported that the chemical states and particle sizes of Pd in the prepared Pd/AC catalysts were significantly influenced by the Pd precursors, Pd(NO_3_)_2_ and Pd(NH_3_)_4_Cl_2_, and thus varied their hydrogenation activity and product selectivity. The 1%Pd/AC (nit) catalyst, prepared using Pd(NO_3_)_2_, presented high performance for selective hydrogenation of poly-FAME into mono-FAME with high oxidation stability, owning to its large Pd particles (8.4 nm). Conversely, the 1%Pd/AC (amc) catalyst, prepared using Pd(NH_3_)_4_Cl_2_, contained small Pd particles (2.7 nm) with a little Cl residues, which could be completely removed by washing with an aqueous solution of 0.1 M NH_4_OH. The small Pd particles gave increased selectivity toward unwanted-FAME components, particularly the saturated fatty acid methyl esters during the hydrogenation of poly-FAME. This selectivity is unprofitable for improving the biodiesel quality.

## 1. Introduction

Nowadays, biodiesel fuel (BDF) is widely used in blends with petroleum diesel. To promote the use of BDF, the blending ratios of BDF with petroleum diesel (labeled B*x*, where *x* indicates the percentage of BDF) have been increased from 3 to 30%, particularly the Association of Southeast Asia Nations (ASEAN) [1]. However, the polyunsaturated fatty acid methyl esters (poly-FAME) in BDF causes its oxidative stability to be low, because the poly-FAME is easily oxidized to peroxides and subsequently acids, and the generated polymerized sludge may damage vehicles [2,3]. Conversely, high contents of saturated fatty acid methyl esters (sat-FAME) worsen the cold flow properties of BDF, and that limits the uses of BDF at low temperatures [4,5,6]. In ASEAN, commercial palm oil-derived biodiesel fuel (palm-BDF) is a major source for blending with petroleum diesel. It generally contains approximately 10% of poly-FAME, mostly methyl linoleate with low oxidation stability (denoted as C_18:2_), approximately 40% of monounsaturated fatty acid methyl esters (mono-FAME), mostly methyl oleate with high oxidation stability and suitable cold flow properties (denoted as C_18:1_), and approximately 50% of sat-FAME, mostly methyl palmitate with low cold flow properties (denoted as C_16:0_) [6]. Therefore, the selective hydrogenation of poly-FAME to corresponding mono-FAME with enhanced oxidation stability and uncompromising cold flow properties and decrease in the amount of obtained sat-FAME have become an imperative for improving the quality of BDF for use in high blends. For example, our research teams recently developed a new catalytic process for selective hydrogenation of commercial palm-BDF over supported metal catalysts, particularly Pd, into partially hydrogenated palm-BDF (denoted as H-FAME), which has low poly-FAME components [4,5,6,7,8,9,10,11]. The high blends of B10-30 fuels formulated by H-FAME (10–30 vol%) with petro-diesel are suitable for diesel-engine vehicles with high safety and security. 

Supported Pd catalysts are commonly used for selective hydrogenation in the refining and petrochemical industry [12,13,14,15,16,17,18,19,20,21]. When Pd catalysts are prepared via aqueous impregnation, the Pd precursor salts and corresponding precursor solutions with different pH values are important factors that could be used to control the interactions (e.g., electrostatic or ion-exchange interactions) between the Pd precursors and supporting materials such as alumina [12,13,14], silica [15,16,17,18,19], and activated carbon (AC) [20,21]. The microporous and mesoporous structures, surface properties, and functional groups of supporting materials also play important roles in controlling the dispersion and chemical state of Pd, hence it influences the performance of obtained Pd catalysts [12,13,14,15,16,17,18,19,20,21,22,23,24,25,26,27,28,29]. The previous study published by Ito et al. reported that the activity of the Cl-containing Pd/Al_2_O_3_ catalyst prepared using a PdCl_2_ precursor salt for naphthalene hydrogenation exceeded that of the Pd/Al_2_O_3_ catalyst prepared using a Pd(NH_3_)_2_(NO_2_)_2_ salt because of a higher metal dispersion [12]. This was attributed to the anion-exchange reaction that occurred between the [PdCl_4_]^2−^ and OH^−^ ions on the Al_2_O_3_ surface (Neo bead-GB Al_2_O_3_, Mizusawa Chemical) during impregnation, which did not occur for the [Pd(NH_3_)_4_]^2+^ cations. Gaspar et al. prepared Pd/γ-Al_2_O_3_ catalysts using PdCl_2_ and Pd(NO_3_)_2_ precursor salts and reported that the complex species of Pd and Cl were formed in the Pd/γ-Al_2_O_3_ catalyst prepared using a PdCl_2_ salt [13]. Due to the dispersion and binding energy (BE) of metallic Pd in the Cl-containing catalyst being higher than those in the Cl-free catalyst, this resulted in the high catalytic activity of the Cl-containing Pd/γ-Al_2_O_3_ catalyst for the hydrogenolysis of methylcyclopentane (MCP) to form 2-methylpentane (2MP), 3-methylpentane, and *n*-hexane. However, the products obtained via the hydrogenolysis of MCP over the Cl-containing Pd/Al_2_O_3_ catalyst contained approximately 12% C_1_–C_5_ hydrocracking products. Similarly, Panpranot et al. reported that the activity of the SiO_2_-supported Pd catalysts prepared using PdCl_2_ as a precursor salt for the complete hydrogenation of 1-hexene to hexane was higher than those of the catalysts prepared using Pd(NO_3_)_2_ or Pd(OOCCH_3_)_2_ as a precursor salt because the Pd particle size of the Cl-containing catalysts was smaller than those of the Cl-free catalysts [15]. Yashnik et al. determined that the pH of the impregnating cationic [Pd(NH_3_)_4_]^2+^ and anionic [PdCl_4_]^2−^ precursor solutions and relative charge of the H-ZSM-5 zeolite surface affected the localization of Pd particles and, consequently, the efficiency and stability of the zeolite-supported Pd catalysts for the hydrodesulfurization of thiophene [18]. 

AC-supported catalysts present several advantages, including large surface area, high stability, inertness in acidic and basic liquid reaction media, and low production cost. Previous studies had demonstrated that AC-supported catalysts delayed the sintering of the active phase and facilitated the recovery of precious metals from spent catalysts via burning off the carbon [30,31,32,33]. It is generally agreed that AC is an attractive supporting material for industrial applications and comparable to silica and alumina. However, previous works have mainly focused on the chemical states of Pd, and the interaction of Pd and alumina (or silica) in the Pd catalysts supported on the alumina (or silica), and further correlated to the performance of these catalysts in the hydro-refining reactions as aforementioned. The effects of the Pd precursor salts on the chemical states and sizes of Pd in AC-supported Pd catalysts, and the performance (e.g., hydrogenation activity and product selectivity) of these catalysts for the hydrogenation of large oil molecules have not been comprehensively understood to date, particularly selective hydrogenation of poly-FAME components in the commercial BDFs, such as palm-BDF [20,21,34,35]. A recent study by Numwong et al., indicated that AC-supported Pd catalysts could catalyze hydrogenation of palm-BDF at 120 °C and 0.4 MPa of H_2_ [34]. However, large Pd particles (10–18 nm) were presented in the AC-supported catalysts, hence it gave low activity of poly-FAME hydrogenation and tended to produce unwanted sat-FAME components with poor cold-flow properties (e.g., the H-FAME samples with high amounts of 72–96% sat-FAME and cloud points of 23–26 °C). This catalytic performance was not profitable for improving the quality of BDF. For catalytic upgrading of commercial BDFs into corresponding H-FAME for use in high blends, particularly commercial palm-BDF widely used in ASEAN, there is still room for the understanding of the chemical state, particle size, and performance of AC-supported Pd catalysts, which are significantly influenced by the nature of Pd precursor salts and AC surface under aqueous impregnation conditions, and their interaction. 

Herein, a commercial AC support was impregnated using two Pd precursor salts: Pd(NO_3_)_2_·*x*H_2_O and Pd(NH_3_)_4_Cl_2_·*x*H_2_O to obtain Cl-free and Cl-containing Pd/AC catalysts. The chemical states and particle sizes of Pd in the prepared Pd/AC catalysts were thoroughly examined by conventional techniques, particularly temperature-programmed reduction (TPR) and X-ray photoelectron spectroscopy (XPS). The influence of Pd particle size and Cl residue on the performance of prepared Pd/AC catalysts were further studied by adding NH_4_Cl into the Cl-free Pd/AC catalyst and by washing the Cl-containing Pd/AC catalyst with NH_4_OH solution. The results of these analyses were correlated with the hydrogenation activity and product selectivity for the partial hydrogenation of commercial palm-BDF to produce corresponding H-FAME. Furthermore, we evaluated the oxidative stability and cold flow properties of the H-FAME samples obtained over the two types of catalysts to compare the effects of the Pd precursor on the catalytic performance of the catalysts.

## 2. Results and Discussion

### 2.1. Characterizations

**Figure 1:**
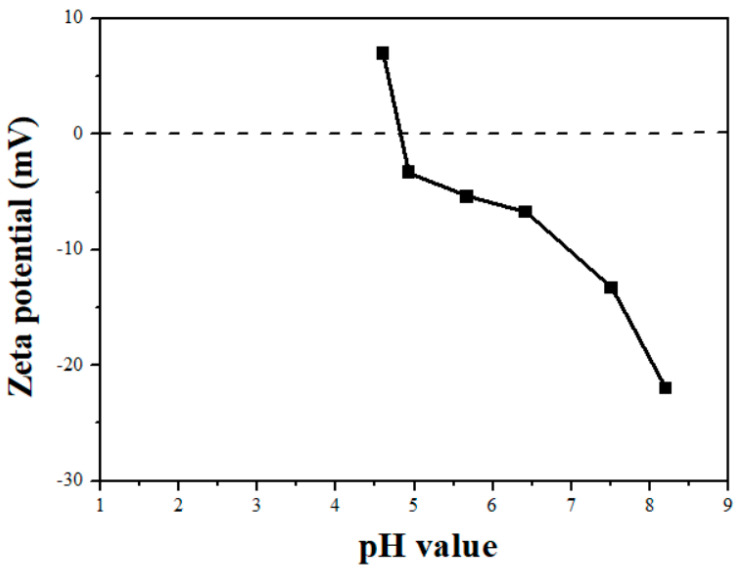
Effect of pH on the surface charge of AC support.

The structural properties of AC and the Pd/AC catalysts are presented in Table 1, in comparison to a commercial Pd/AC catalyst with a Pd loading of 5 wt.% (termed 5%Pd/AC (com), which is used as a reference catalyst). The Brunauer–Emmett–Teller specific surface area (*S_BET_*), pore diameter (*D_p_*), and total pore volume (*V_total_*) of AC were 1782 m^2^ g^−1^, 0.87 nm, and 0.70 cm^3^ g^−1^, respectively. In addition, the point of zero charge (PZC) of AC was 4.8, as illustrated in Figure 1. When the Pd(NO_3_)_2_ and Pd(NH_3_)_4_Cl_2_ precursors were dissolved in deionized water, the resulting aqueous solutions contained [Pd(H_2_O)_4_]^2+^ and [Pd(NH_3_)_4_]^2+^ cations, respectively. The pH values of the Pd(NO_3_)_2_ and Pd(NH_3_)_4_Cl_2_ precursor solutions ranged between 1.57–1.60 and 8.78–8.80, respectively.

The surface charge of AC was positive at pH < 4.8 and negative at pH > 4.8. Therefore, the interactions between the [Pd(NH_3_)_4_]^2+^ cations and negatively charged AC were expected to be strong. Conversely, the impregnation of the [Pd(H_2_O)_4_]^2+^ cations on positively charged AC at low pH levels led to weak interactions between the Pd precursor ions and AC surface. Solar et al. [36] and Banerjee et al. [37] indicated that the strong interactions between the Pd cations and AC hindered the growth and mobility of Pd particles in the calcined and reduced samples.

After Pd impregnation, the Pd loadings of the 1%Pd/AC (nit) and 1%Pd/AC (amc) catalysts were 0.9 and 1.0 wt.%, respectively, which were close to the theoretical values (Table 1). The Cl species were detectable in the 1%Pd/AC (amc) catalyst, and the Cl loading was 0.1 wt.% even after the catalyst was reduced with H_2_ at 300 °C. The acidities of the 1%Pd/AC (nit) and 1%Pd/AC (amc) catalysts were 0.10 and 0.12 mmol g^−1^, respectively, which were slightly higher than that of AC. This was ascribed to the formation of weakly acidic surface oxygenated species, such as –OH groups, via impregnation [38]. The *S_BET_*, *D_p_*, and *V_total_* values of the prepared Pd catalysts were slightly lower than those of AC, owing to the surface reactions of the Pd precursors with AC. However, all prepared Pd catalysts still presented high *S_BET_* values in the range of 1750–1780 m^2^ g^−1^, which were similar to that of AC and were higher than that of 5%Pd/AC (com). This indicated that the effect of the impregnation pH on the structural properties of AC was negligible, and the structural property of prepared Pd catalysts was better than that of a 5%Pd/AC (com).

**Figure 2:**
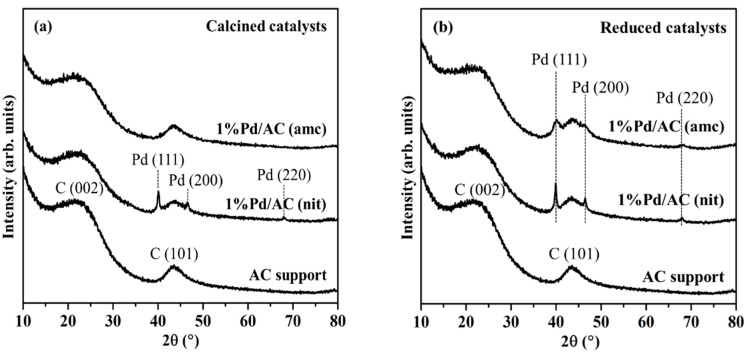
XRD patterns of (**a**) calcined and (**b**) reduced 1%Pd/AC (nit) and 1%Pd/AC (amc) catalysts and AC support.

Figure 2a,b depict the XRD patterns of the calcined and reduced catalysts, respectively. The results were compared with that of the AC support. For the AC support, the characteristic peaks of amorphous carbon frameworks were observed at 2*θ* = 23.5° and 43.6° [39]. For the calcined 1%Pd/AC (nit) catalyst, three intense peaks were observed at 40.1°, 46.6°, and 68.1° corresponding to the (111), (200), and (220) planes of face-centered cubic Pd (PDF# 46-1043), respectively. However, no Pd species were observed in the XRD pattern of the calcined 1%Pd/AC (amc) catalyst, which suggested that the X-ray–amorphous Pd particles were highly dispersed on the AC surface. The presence of several distinct peaks of metallic Pd in the XRD patterns of the reduced 1%Pd/AC (nit) and 1%Pd/AC (amc) catalysts indicated the reduction of PdO to metallic Pd particles, which presented increased crystallinity. However, the diffraction peaks of 1%Pd/AC (nit) are still stronger and narrower than those of 1%Pd/AC (amc), indicating the Pd particles of the reduced 1%Pd/AC (nit) catalyst are larger than that of the reduced 1%Pd/AC (amc) catalyst. 

**Figure 3:**
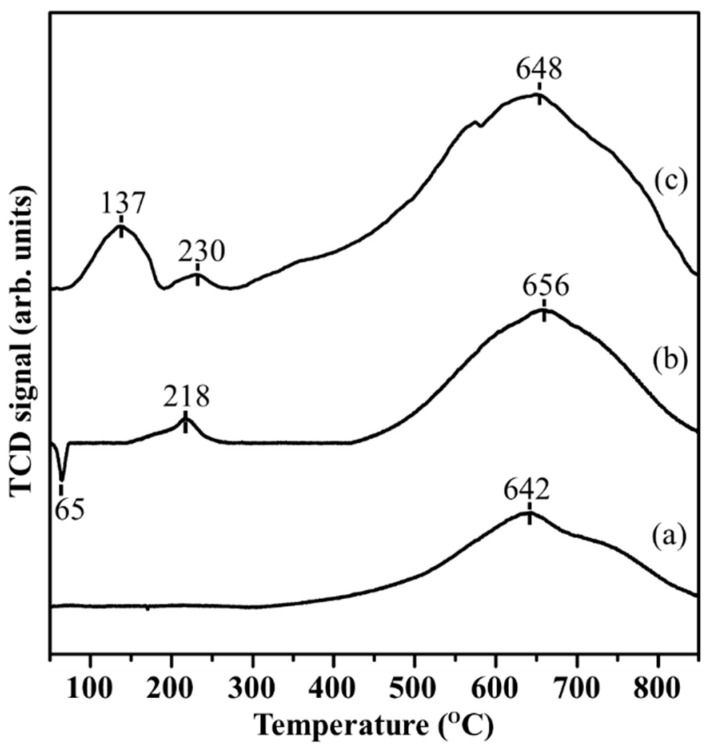
H_2_-TPR profiles of (**a**) AC support, (**b**) 1%Pd/AC (nit), and (**c**) 1%Pd/AC (amc) catalysts.

The H_2_-TPR profiles of AC and the 300 °C N_2_-calcined 1%Pd/AC (nit) and 1%Pd/AC (amc) catalysts are illustrated in Figure 3. All samples presented a broad peak centered at approximately 640–660 °C. This was ascribed to the thermal instability of AC, which formed gas products such as CH_4_, CO, and CO_2_ via the methanation and thermal decomposition reactions of microporous carbon framework under a reducing atmosphere (Supplementary Figure S1) [40,41,42,43]. The peaks at high temperature in the H_2_-TPR profiles of the prepared Pd catalysts were stronger than that of AC, and that was attributed to the dissociation of H_2_ being facilitated over Pd nanoparticles and spilled to the surface of AC (i.e., the spillover effect). Moreover, the number of oxygenated groups on the surface of the Pd catalysts might be higher than that on the surface of AC owing to the impregnation of the acidic Pd precursors. The reactions of the surface oxygenated groups with the spillover H_2_ species resulted in the strong peaks in the 640–660 °C range. However, the prepared Pd catalysts were entirely stable under mild reaction conditions (<100 °C), as described in Section 2.2.

The H_2_ consumption peak in the H_2_-TPR profile of 1%Pd/AC (nit) at 218 °C (curve (b) in Figure 3) was associated with the reduction of the Pd^2+^ species derived from the strong interaction of the PdO(NO*_x_*) residues with the oxygenated groups on the AC surface. A sharp peak attributed to the release of H_2_ was observed at 65 °C, and was ascribed to the recombination of the hydride on *β*-PdH and desorption to H_2_ [44]. This hypothesis was supported by the XRD pattern of the calcined 1%Pd/AC (nit) catalyst (Figure 2a), which indicated that metallic Pd species were present in the calcined 1%Pd/AC (nit) catalyst and were nearly unchanged, after reduction. In contrast, two H_2_ consumption peaks were observed in the H_2_-TPR profile of 1%Pd/AC (amc) (curve (c) in Figure 3). The peak at 137 °C was associated with the reduction of PdO nanoparticles impregnated on AC. The data in Table 1 suggest that a large amount of CO could be adsorbed on 1%Pd/AC (amc), which indicated that this catalyst contained highly dispersed Pd particles with an average size of 0.8 nm. The Pd particle size was similar to the pore size of AC. It speculates that the small Pd particle may stay in the inner pores of AC. The peak at 230 °C could be related to the reduction of Pd^2+^ species (presumably PdOCl*_x_*, which interacted strongly with the oxygenated groups on the surface of AC) to metallic Pd species. This behavior was similar to that of 1%Pd/AC (nit) and has been previously reported in the literature [45,46,47]. The Cl residues generally form in metal catalysts prepared using Cl-containing precursors and may affect the catalytic properties of the catalysts.

**Figure 4:**
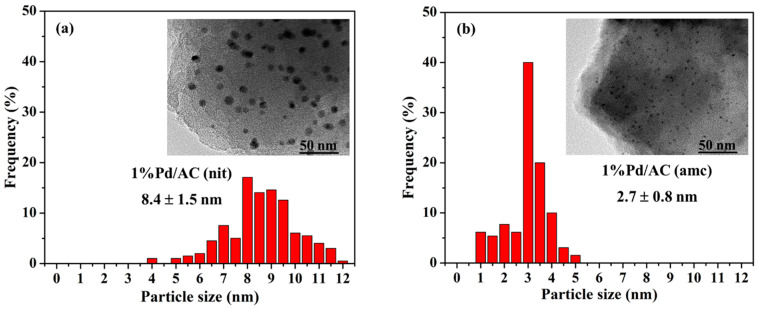
Pd size distribution and TEM micrographs of the (**a**) 1%Pd/AC (nit) and (**b**) 1%Pd/AC (amc) catalysts.

The TEM images of the prepared Pd catalysts revealed that the 1%Pd/AC (nit) catalyst contained large Pd particles (8.4 nm) with a relatively broad size distribution (±1.5 nm), and the 1%Pd/AC (amc) catalyst consisted of small Pd particles (2.7 nm) with a relatively narrow size distribution (±0.8 nm) (Figure 4a,b, respectively). The high dispersion of the 1%Pd/AC (amc) catalyst could be attributed to the strong interactions between the [Pd(NH_3_)_4_]^2+^ cations and AC support, whereas the opposite was true for the 1%Pd/AC (nit) catalyst. Although the small Pd particles were indiscernible in the TEM images of the 1%Pd/AC (amc) catalyst, the CO chemisorption data suggested that the 1%Pd/AC (amc) catalyst contained small Pd particles (<1 nm). These particles could be associated with the 137 °C peak in the H_2_-TPR profile of 1%Pd/AC (amc).

**Figure 5:**
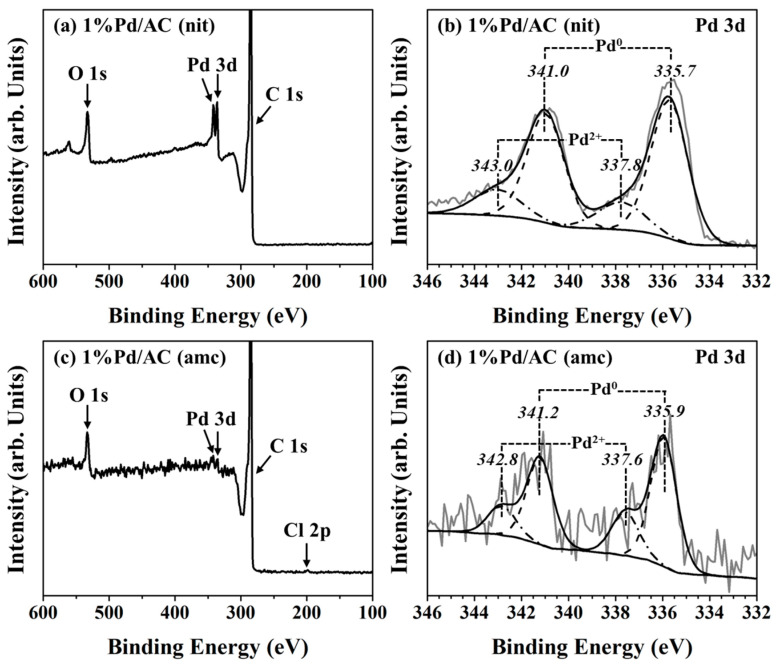
XPS analysis of the chemical states of Pd in (**a**) and (**b**) 1%Pd/AC (nit), and (**c**) and (**d**) 1%Pd/AC (amc).

XPS analysis was used to study the surface composition and chemical states of Pd and residual elements in the Pd/AC catalysts, which were calcined at 300 °C in N_2_ and reduced at 300 °C in H_2_ prior to the measurement. The wide-range XPS spectra of 1%Pd/AC (nit) and 1%Pd/AC (amc) are illustrated in Figure 5a,c, respectively. The O 1*s*, C 1*s*, and Pd 3*d* peaks were observed in the XPS profiles of the prepared Pd/AC catalysts. In addition, the characteristic 197.7 eV peak of Cl 2*p* was slightly visible in the XPS profile of the 1%Pd/AC (amc) catalyst (Figure 5c) and indicated the presence of a little Cl residues on its surface. Furthermore, this implied that owing to the relatively strong interaction between the Pd^2+^ cations and highly electronegative Cl^−^ anions, Cl could not be completely eliminated from the impregnated Pd species via calcination and subsequent reduction. In contrast, no N species were observed in the XPS profile of 1%Pd/AC (nit), which indicated that most N species could be easily removed from the 1%Pd/AC (nit) catalyst via calcination or reduction.

The Pd 3*d*_5/2_ and 3*d*_3/2_ peaks of metallic Pd can be observed at around 336 and 341 eV, respectively, and those of the Pd^2+^ cations can be observed at around 338 and 343 eV [48,49,50]. The reduced 1%Pd/AC (nit) and 1%Pd/AC (amc) catalysts contained metallic Pd particles (major species) and Pd^2+^ species (minor species). The 3*d*_5/2_ peak of Pd^0^ in the XPS profiles of 1%Pd/AC (nit) and 1%Pd/AC (amc) were located at around 336 eV, and were upshifted by around 1 eV compared with those of bulk metallic Pd (BE = 335.0 eV). The slight higher BE of the prepared Pd catalysts suggested that Pd was electron-deficient (Pd^δ+^) owing to the co-existence of Pd and PdO species for 1%Pd/AC (nit) and the co-existence of Pd and PdO species with a little Cl residues for 1%Pd/AC (amc) [51]. The ratios of the Pd^0^ and Pd^2+^ peak areas are summarized in Table 1 and are correlated with the chemical states and sizes of Pd in the prepared Pd catalysts. The Pd^0^/Pd^2+^ ratio of the 1%Pd/AC (nit) catalyst with large Pd sizes (approximately 8 nm) was 3.6, which was significantly larger than that of the 1%Pd/AC (amc) catalyst (Pd^0^/Pd^2+^ = 2.8) with small Pd particles (approximately 3 nm). A fraction of the Pd^2+^ cations were formed by exposing the metallic Pd particles to air, particularly 1%Pd/AC (amc), when the reduced catalyst samples were transferred to the ex situ cell for XPS analysis. Noted that the Pd^2+^ cations in the XPS profile of the 1%Pd/AC (amc) catalyst was presumably related to the PdO species with a little Cl residues, such as PdOCl_2_ species [52,53]. The Cl/Pd weight ratio of the 1%Pd/AC (amc) catalyst was only 0.11, which was much lower than the theoretically expected ratio of PdOCl_2_ (0.67). Consequently, the Pd^2+^ cations in the 1%Pd/AC (amc) catalyst originated from a mixture of PdO (major contributor) and PdOCl_2_ (minor contributor). The signals at around 198 and 201 eV in the XPS profile of 1%Pd/AC (amc) were attributed to the Pd–Cl and C–Cl bonds, respectively (Supplementary Figure S2), and indicated again the presence of a little Cl residues on the AC surface [50].

It is specially noted that the Pd 3d XPS signals of 1%Pd/AC (amc) catalyst with small Pd particles (high Pd dispersion) were less intense with relatively low signal-to-noise ratio than those of 1%Pd/AC (nit) catalyst with large Pd particles (low dispersion). Since XPS is a surface-sensitive technique, the decrease in the Pd 3d XPS signals of 1%Pd/AC (amc) catalyst and the low signal-to-noise ratio are associated with the interference of carbon framework (e.g., the small Pd particles (approximately 3 nm) were partially covered by the microporous carbon framework). By contrast, large Pd particles (approximately 8 nm) were presented at the pore mouths or outer surfaces of 1%Pd/AC (nit) catalyst. Hence, they gave intense Pd 3d XPS signals with high signal-to-noise ratio, and displayed high activity/selectivity in the synthesis of H-FAME due to high accessibility as discussed in Section 2.2.

### 2.2. Partial Hydrogenation of Palm-BDF to Corresponding H-FAME

We evaluated the hydrotreating performance of the 1%Pd/AC (nit) and 1%Pd/AC (amc) catalysts for the partial hydrogenation of a commercial palm-BDF, which contained 8.6 wt.% of poly-FAME (mostly C_18:2_), to produce H-FAME, which had low poly-FAME components. The poly-FAME components of palm-BDF consisted mostly of methyl linolenate (C_18:3_; 0.1 wt.%) and methyl linoleate (C_18:2_; 8.5 wt.%). The major mono-FAME components of palm-BDF were *cis*-methyl oleate (*cis*-C_18:1_; 36 wt.%) and *trans*-methyl oleate (*trans*-C_18:1_; 0.2 wt.%), and the major sat-FAME components of palm-BDF were methyl palmitate (C_16:0_; 45.5 wt.%) and methyl stearate (C_18:0_; 4.8 wt.%). The FAME composition of palm-BDF was similar to that reported in the literature [54]. The relative autoxidation rates of C_18:1_, C_18:2_, and C_18:3_ were reported to be 1, 41, and 98, respectively [55]. In this study, we aimed to partially hydrogenate the poly-FAME components of palm-BDF into corresponding mono-FAME components. However, *trans*-C_18:1_, which presents relatively poor cold flow properties, was easily formed via the isomerization of *cis*-C_18:1_ with good cold flow properties during the partial hydrogenation of palm-BDF to H-FAME over Pd catalysts and affected the quality of H-FAME. It is due to that the melting point of *trans*-C_18:1_ is approximately 10 °C, which is much higher than that of *cis*-C_18:1_ (−20 °C). On the other hand, the complete hydrogenation of the poly-FAME components to corresponding C_18:0_ components with a melting point of approximately 38 °C also occurred and caused the resulting H-FAME to present poor cold flow properties. To improve oxidation stability without sacrificing the cold flow properties, the selective hydrogenation of C_18:3_ and C_18:2_ to *cis*-C_18:1_ was favored, and the formation of *trans*-C_18:1_ and sat-C_18:0_ should be prevented, particularly at the high poly-FAME conversion (≥90%). A good Pd catalyst should give high performance (i.e., both activity and selectivity) for conversion of poly-FAME components into corresponding mono-FAME components, particularly the *cis*-mono-FAME, and minimize the unwanted products, such *trans*-mono-FAME and sat-FAME. With decreasing the poly-FAME components and minimizing the formation of *trans*-mono-FAME and sat-FAME components, H-FAME with enhanced quality can be a new type of palm-BDF appropriate for high blends, particularly in ASEAN.

**Figure 6:**
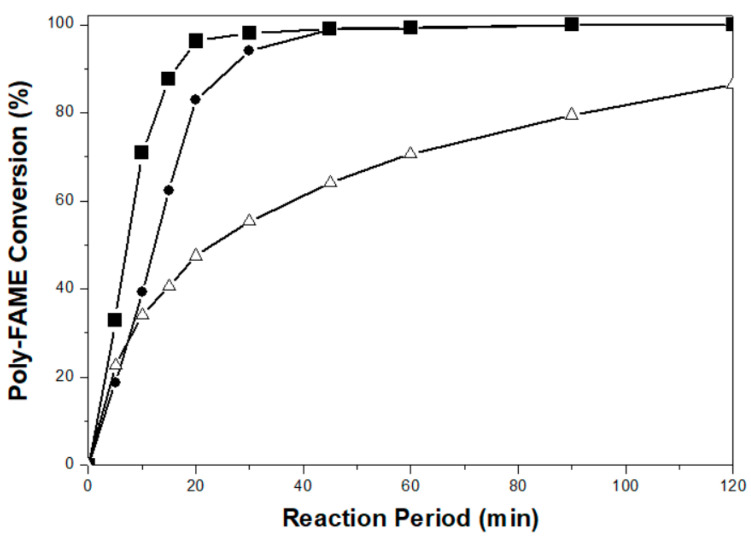
Poly-fatty acid methyl esters (FAME) conversion dependence of reaction time over: (■) 1%Pd/AC (nit), (●) 1%Pd/AC (amc), and (△) 5%Pd/AC (com) catalysts.

Figure 6 presents the time evolution of the conversion of the poly-FAME components (including C_18:3_ and C_18:2_) over the prepared 1%Pd/AC (nit) and 1%Pd/AC (amc) catalysts, in comparison to a reference catalyst of 5%Pd/AC (com). The 1%Pd/AC (nit) catalyst with large Pd particles (8.4 nm) and no N contaminants could convert the poly-FAME components quickly (<60 min), whereas the 1%Pd/AC (amc) catalyst with small Pd particles (2.7 nm) and a little Cl residues (0.1 wt.%) required longer times to achieve complete conversion of the poly-FAME components. A reference catalyst of 5%Pd/AC (com) with small Pd sizes (3.3 nm) was unable to completely convert poly-FAME into corresponding mono- and sat-FAME even after a long reaction time of 120 min, presumably due to slow molecular diffusion through the microporous framework with a relatively low surface area and pore volume. The initial rates were calculated by dividing the molar amount of poly-FAME components consumed by the mass of Pd at the first 5 min of the reaction. The initial rates of the analyzed catalysts decreased as follows: 1%Pd/AC (nit) > 5%Pd/AC (com) > 1%Pd/AC (amc) (Table 2), indicating the 1%Pd/AC (nit) catalyst gave the highest activity in partial hydrogenation of a commercial palm-BDF.

**Figure 7:**
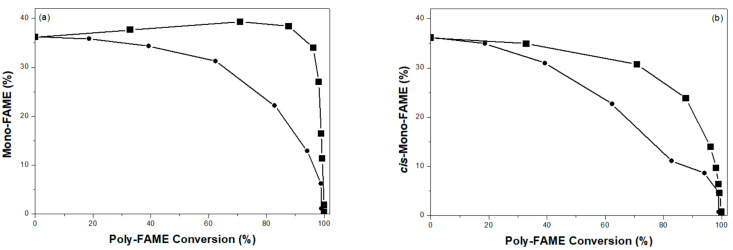

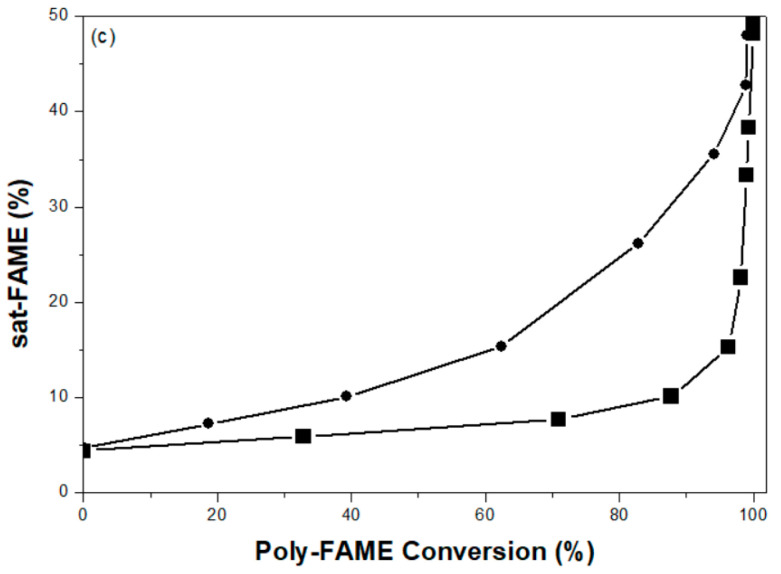
Percentages of (**a**) mono-FAME, (**b**) *cis*-mono-FAME, and (**c**) sat-FAME as functions of the conversion of poly-FAME components over: (■) 1%Pd/AC (nit) and (●) 1%Pd/AC (amc).

Figure 7 presents the amounts of mono-FAME (a total amount of mono-C_18:1_), *cis*-mono-FAME (a total amount of *cis*-mono-C_18:1_), and sat-FAME (a total amount of sat-C_18:0_) as a function of the poly-FAME conversion over the prepared Pd catalysts. The 1%Pd/AC (nit) catalyst presented high selectivity toward the mono-C_18:1_ FAME at the high poly-FAME conversion (>90%), particularly the *cis*-form, whereas the opposite was true for the sat-C_18:0_ FAME. While the study on the selectivity of 5%Pd/AC (com) was failed because of its poor activity in partial hydrogenation of a commercial palm-BDF. On the other hand, the 1%Pd/AC (amc) catalyst presented very poor selectivity for the mono-FAME and high selectivity for sat-FAME, and it favored the complete hydrogenation of palm-BDF. These results speculated that the selectivity of 1%Pd/AC (nit) catalyst in partial hydrogenation of a commercial palm-BDF was also higher than that of 1%Pd/AC (amc) catalyst.

The activity of 1%Pd/AC (nit) and 1%Pd/AC (amc) catalysts was further evaluated using their turnover frequency (TOF) values, which were calculated at the first 5 min of reaction using Equation (1). The selectivity was further evaluated by the *cis*-/*trans*-C_18:1_ ratio and degree of complete hydrogenation at the poly-FAME conversion of 90%. The degree of complete hydrogenation was determined using Equation (2):

(1) $${\mathrm{TOF}\;(h}^{-}{}^{1})\;=\;\left(\frac{\frac{C18:2\;\mathrm{conversion}\;\left(\%\right)}{100}\;\times \;\mathrm{Amount}\;\mathrm{of}\;\mathrm{biodiesel}\;\left(g\right)\;\times \;\frac{1}{\mathrm{Mw}\;\mathrm{of}\;C18:2\;\left(294\;g/\mathrm{mol}\right)}}{\frac{\%\;\left(m/m\right)\;\mathrm{Pd}}{100}\;\times \;\frac{\%\;\mathrm{Pd}\;\mathrm{dispersion}}{100}\;\times \;M_{\mathrm{catalyst}}\;\left(g\right)\;\times \;\frac{1}{\mathrm{Mw}\;\mathrm{of}\;\mathrm{Pd}}}\right)÷\mathrm{time}$$

and

(2) $$\mathrm{Degree}\;\mathrm{of}\;\mathrm{complete}\;\mathrm{hydrogenation}\;\left(\%\right)\;=\;\left(\frac{C18:0\;\mathrm{in}\;\mathrm{product}-C18:0\;\mathrm{in}\;\mathrm{feed}}{\mathrm{Total}\;\mathrm{amount}\;\mathrm{of}\;C18:1\;\mathrm{in}\;\mathrm{product}}\right)$$

where Mw is the molecular weight and M_catalyst_ is the mass of the catalyst.

Again, the TOF of 1%Pd/AC (nit) catalyst was much higher than that of 1%Pd/AC (amc) and 5%Pd/AC (com) catalysts, speculating that large Pd particles (approximately 8 nm) were superior to small Pd particles (approximately 3 nm) in catalyzing the partial hydrogenation of a commercial palm-BDF. This observation is similar to the recent study on the partial hydrogenation of soybean oil-derived BDF into corresponding H-FAME using the SiO_2_-supported Pd catalysts with relatively large Pd particles (6–8 nm) [56]. When the poly-FAME conversion was 90%, the *cis*-/*trans*-C_18:1_ ratio of 1%Pd/AC (nit) catalyst was 1.4, and it was similar to that of 1%Pd/AC (amc) catalyst, indicating that the 3–8 nm Pd particles display similar performance (i.e., both activity and selectivity) in the isomerization of *cis*-mono-FAME to *trans*-mono-FAME. However, the degree of complete hydrogenation for the 1%Pd/AC (nit) catalyst was much lower than that of the 1%Pd/AC (amc) catalyst. It speculated that large Pd particles (approximately 8 nm) presented high selectivity for the mono-FAME, while the reverse was true for small Pd particles (approximately 3 nm), which presented high selectivity for sat-FAME and favored the complete hydrogenation of palm-BDF. The used catalysts were studied by XRD, although they gave relatively poor XRD signals with low signal-to-noise ratio due to the contamination of residuals oils (Supplementary Figure S3). The results depicted that the diffraction peaks of Pd species presented in the used Pd catalysts were similar to those in the as-prepared Pd catalysts, indicating that the Pd sizes of Pd/AC(nit) and Pd/AC(amc) catalysts were slightly influenced by the studied reaction conditions.

The hydrogenation of poly-FAME to corresponding mono-FAME and sat-FAME is a complex process that involves hydrogenation and isomerization, as illustrated below [57]:
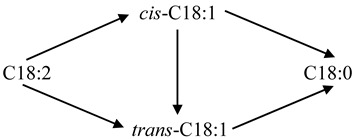
(3)
which can be simplified as follows:
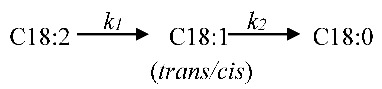
(4)

The *k*_1_ and *k*_2_ rate constants were calculated using a first-order kinetic model [58], and the obtained values are listed in Table 2. Saturation selectivity (*S*) was defined as the *k*_1_/*k*_2_ ratio, and the *S* values of the catalysts in this study are listed in Table 2. The *k*_1_ and *k*_2_ values of the 1%Pd/AC (nit) catalyst were high (0.23 s^−1^ g^−1^) and low (0.091 s^−1^ g^−1^), respectively, which resulted in a large *S* value of 2.6. Conversely, the *k*_1_ and *k*_2_ values of the 1%Pd/AC (amc) catalyst were small and high, respectively, and the corresponding *S* value was smaller than 1. The low *S* value of the 1%Pd/AC (amc) catalyst indicated that the rates of adsorption and hydrogenation of poly-C_18:2_ over the small and Cl-containing Pd particles (approximately 3 nm, 0.1 wt.% of Cl) were hindered, whereas the opposite was true for deep hydrogenation of mono-C_18:1_.

For deep understanding of the effects of Pd particle size and Cl residue, the post-treated catalysts of 1%Pd/AC (nit)-Cl and 1%Pd/AC (amc)-noCl were studied in the partial hydrogenation of a commercial palm-BDF under the same reaction conditions (Supplementary Figure S4). The 1%Pd/AC (nit)-Cl catalyst with approximately 0.1 wt.% of Cl was prepared by impregnation of NH_4_Cl aqueous solution on the dried 1%Pd/AC (nit) catalyst. The 1%Pd/AC (amc)-noCl catalyst was obtained by washing the 1%Pd/AC (amc) catalyst with 0.1 M of NH_4_OH solution, and the XPS analysis indicated that the Cl residue could be completely removed. The detailed procedures were described in the Supplementary Materials. The results presented that the activity of Cl-containing Pd catalysts (1%Pd/AC (nit)-Cl and 1%Pd/AC (amc)) was slightly higher than those of Cl-free Pd catalysts (1%Pd/AC (nit) and 1%Pd/AC (amc)-noCl) (Supplementary Table S1). However, the product selectivity for mono-FAME, *cis*-mono-FAME, and sat-FAME as a function of reaction time over Cl-containing and Cl-free Pd catalysts remained nearly unchanged, particularly those related to high poly-FAME conversions. This result indicated that the hydrogenation of poly-FAME components over the Cl-containing Pd particles was slightly facilitated, presumably due to the Pd surface being slightly electron-deficient owing to the high electronegativity of the Cl species [56,59,60,61]. However, the activity and selectivity of Pd/AC catalysts in the partial hydrogenation of a commercial palm-BDF were predominately influenced by the sizes of Pd particles. The large Pd particles (approximately 8 nm) of 1%Pd/AC(nit) catalyst and corresponding Cl-containing counterpart were superior to the small Pd particles (approximately 3 nm) of 1%Pd/AC (amc) catalyst and corresponding Cl-free counterpart in catalyzing the partial hydrogenation of a commercial palm-BDF. Moreover, the selective hydrogenation of poly-C_18:2_ over the large Pd particles could be facilitated, whereas the formation of sat-C_18:0_ was limited. It should be associated with high accessibility of large Pd particles on the AC surfaces, which facilitated adsorption and hydrogenation of poly-C_18:2_, and subsequently desorption of forming mono-C_18:1_. As a result, the large initial rate, TOF and *S* value of 1%Pd/AC (nit) catalyst with large Pd particles (approximately 8 nm) presented the high hydrotreating performance in the synthesis of H-FAME. The catalytic performance of the 1%Pd/AC (nit) catalyst was comparable to that of the recently reported Pd/SBA-15 catalyst, which presented high *k_1_* and low *k_2_* values for the synthesis of H-FAME owing to the Pd nanoparticles being impregnated on the surface of C-coated SBA-15 [54]. Because SBA-15 presented low acid capacity and hydrophobic surface, its affinity toward the methylene bonds of the poly-FAME components was high. Consequently, the desorption of the mono-FAME components over Pd/SBA-15 was enhanced. However, the recycling of precious Pd metal from spent Pd/SBA-15 catalysts will be a difficult task. It is a fact that SBA-15 is a mesoporous silica material, and precious Pd metal cannot be simply recycled from spent Pd/SBA-15 catalysts by burning. 

### 2.3. FAME Composition and Fuel Properties

In Table 3, the FAME composition (e.g., C_18:3_, C_18:2_, C_18:1_, and C_18:0_) and fuel properties of the H-FAME obtained over the prepared Pd/AC catalysts at a poly-FAME conversion of 90% were compared with those of the palm-BDF. The H-FAME obtained over 1%Pd/AC (nit) contained 0.9 wt.% of C_18:2_-FAME, 37.8 wt.% of C_18:1_-FAME, and 10.9 wt.% of C_18:0_-FAME components, corresponding to the poly-, mono-, and sat-FAME components with an 18-carbon chain, respectively. The *cis-*/*trans*-C_18:1_ ratio was 1.4 (also see Table 2). By contrast, the H-FAME obtained over 1%Pd/AC (amc) contained a low amount of C_18:1_-FAME components and a high amount of C_18:0_-FAME component while the *cis*-/*trans*-C_18:1_ ratio was slightly varied to 1.3. It is another indication that the Pd particle sizes (3–8 nm) are insensitive to the isomerization of *cis*-mono-FAME components to *trans*-counterparts. However, large Pd particles (8 nm) give higher activity in partial hydrogenation of poly-FAME components and higher selectivity toward mono-FAME components than small Pd particles (3 nm). Although the Pd catalysts with relatively low structural property and high Pd loading were used in the synthesis of H-FAME under severe conditions [34], the previously reported carbon-supported Pd catalysts (e.g., 2%Pd/AC and 2%Pd/CA) with larger Pd particles (10–18 nm) gave high selectivity toward sat-FAME, which was unprofitable for improving the biodiesel quality. It can be said that the Pd/AC (nit) catalyst with approximately 8 nm Pd particles is a highly active and selective catalyst for the synthesis of H-FAME under the studied reaction condition (80 °C, 0.5 MPa of H_2_, 1 g of catalyst and 150 g of oil).

The poly-FAME, mono-FAME, and sat-FAME components of the H-FAME obtained over the prepared Pd/AC catalysts are also listed in Table 3 and correlated to the oxidation stability and cloud point. The results are further compared to those of the H-FAME obtained by carbon-supported Pd catalysts in the literature [34]. The oxidation stability of the antioxidant-stabilized palm-BDF as a commercial product is 13 h, which is higher than that of the antioxidant-free palm-BDF (<5 h) [60,61]. The oxidation stability of palm-BDF is higher than those specified in the ASTM D6751 international fuel standard (>3 h), EN 14,214 international fuel standard (>8 h), and Thai standard (>10 h) for the formulation of B7 fuels [62,63]. It is a fact that the palm-BDF used in this study was a commercial Thai product; therefore, its quality must meet the specifications of the Thai standard. However, as mentioned in the introduction, we aimed to increase the oxidation stability of commercial palm-BDF so that the upgraded palm-BDF (i.e., H-FAME) could be used to formulate high blends, such as B10–B30. Table 3 shows that the H-FAME obtained over all the catalysts in this study presented a high oxidation stability of 60 h owing to most of the poly-FAME components being hydrogenated. However, the H-FAME obtained over the 1%Pd/AC (amc) catalyst presented a high cloud point owing to the presence of a large amount of sat-FAME in its composition, and would be unsuitable for blending with petrodiesel, particularly for the Chiang Mai area in Thailand (the standard cloud point in Thailand is 16 °C). The H-FAMEs obtained by the previously reported carbon-supported Pd catalysts (e.g., 2%Pd/AC and 2%Pd/CA) [34] showed poor oxidation stability and cold flow properties caused by their improper compositions of mono- and sat-FAME. Conversely, the H-FAME obtained over the 1%Pd/AC (nit) catalyst presented high oxidation stability without sacrificing their cold flow properties (the cloud point of 18 °C of this sample was closed to the Thai national standard) owing to the poly-FAME components being selectively hydrogenated to corresponding mono-FAME, and over-hydrogenation being minimized. The cloud point of H-FAME could be reduced to below 16 °C via further purification/separation processes, such as precipitation or distillation, to remove the saturated FAME components [64,65]. Although the reaction parameters should be further studied on a pilot plant before industrialization, the 1%Pd/AC (nit) catalyst with large Pd particles presented high activity and suitable selectivity toward monounsaturated FAME. Consequently, 1%Pd/AC (nit) would be a good candidate for large scale synthesis of H-FAME, owing to its low operation cost and facile Pd metal recycling via burning the spent catalyst.

## 3. Materials and Methods

### 3.1. Materials

Granular AC (*S_BET_* value of 1593 m^2^ g^−1^ and particle size of 30–60 mesh) with a low amount of metal impurities was purchased from Kuraray Company Limited (Chiyoda-Ku, Japan), and was used as support. The characteristics of the AC are summarized in Table 1. Pd(NO_3_)_2_·*x*H_2_O (≥99.8 wt.%, Alfa Aesar, Tewksbury, MA, USA) and Pd(NH_3_)_4_Cl_2_·*x*H_2_O (Pd content of 40.16 wt.%, N. E. Chemcat Corporation, Tokyo, Japan) were used as Pd precursors. A commercial Pd/AC catalyst (i.e., 5%Pd/AC (com)) with a Pd loading of 5 wt.% was purchased from Fujifilm Wako Pure Chemical Corporation (Tokyo, Japan), and used as a reference Pd catalyst. The *S_BET_*, *D_P_*, and *V_total_* values were 919 m^2^ g^−1^, 3.2 nm, and 0.77 cm^3^ g^−1^, respectively, which were lower than those of prepared Pd/AC (nit) and Pd/AC (amc) catalysts. The Pd size and dispersion were 3.3 nm and 15%, respectively, which were between the values of prepared Pd/AC (nit) and Pd/AC (amc) catalysts. Palm-BDF was kindly supplied by Global Green Chemical Public Company Limited (Bangkok, Thailand) and was used as biodiesel feedstock for partial hydrogenation in this study.

### 3.2. Preparation of the Pd/AC Catalysts

Before impregnation, AC was dried at 110 °C. Two Pd/AC catalysts with Pd loadings of 1 wt.% were prepared via the impregnation of Pd aqueous solutions on AC under vacuum (approximately 0.1 torr) at 25 °C followed by aging for 24 h. The Pd aqueous solutions were obtained by dissolving 0.022 g of Pd(NO_3_)_2_·*x*H_2_O and 0.025 g Pd(NH_3_)_4_Cl_2_·*x*H_2_O precursors in 0.4 mL of deionized water. The samples were subsequently dried at 60 °C for 6 h using a rotary evaporator followed by calcination at 300 °C in a N_2_ flow for 2 h. The prepared samples were labeled 1%Pd/AC (nit) and 1%Pd/AC (amc), where (nit) and (amc) indicate that Pd(NO_3_)_2_·*x*H_2_O and Pd(NH_3_)_4_Cl_2_·*x*H_2_O were used as precursors, respectively. Prior to using the catalysts for the partial hydrogenation of palm-FAME, they were reduced at 300 °C for 1 h under a H_2_ flow of 50 mL min^−1^.

### 3.3. Characterizations

The PZC of carbon materials can be measured using a simplified version of the pH shift method [25]. The surface charge of AC was determined by measuring its zeta potential over a wide pH range using a Zetasizer Nano ZS (Malvern instrument, Malvern, UK) instrument, equipped with an MPT-2 (Malvern instrument, Malvern, UK) multi-purpose titrator. The solution pH was adjusted to the target pH using 0.1 M HCl or NaOH. The reducibility of the prepared Pd catalysts was determined using the TPR technique utilizing a BELCAT-B (BEL, Osaka, Japan) chemisorption analyzer, equipped with a thermal conductivity detection (TCD) instrument and a BELMass mass spectrometry (MS) apparatus. The samples were pretreated at 120 °C for 30 min under an Ar stream to eliminate the physically adsorbed water followed by purging with an Ar stream at 40 °C until the TCD signal was stable. For the TPR measurements, the TCD signals were recorded in the temperature range of 40–900 °C at a ramping rate of 10 °C min^−1^ under a 5 vol.% H_2_/Ar flow. The MS signals of H_2_, CH_4_, CO, and CO_2_ were monitored by the m/z ratios of 2, 16, 28, and 44, respectively. Note that the CO and CO_2_ were formed by thermal decomposition of AC, while CH_4_ was formed by methanation of AC (Supplementary Figure S1) [40,41,42,43]. The powdered X-ray diffraction (XRD) patterns of the catalysts were recorded using an X’Pert PRO (PANalytical, Netherlands) XRD system with Cu Kα radiation (*λ* = 1.5406 Å), in the 2*θ* range of 10–80° at a scanning rate of 0.04° s^−1^. An ARL Perform’X (Thermo Fisher Scientific, Bartlesville, OK, USA) wavelength dispersive X-ray fluorescence spectroscope equipped with an X-ray tube with a Rh anode and operated at 70 kV and 140 mA was used to analyze the Pd and Cl contents of the prepared Pd/AC catalysts. The surface area and porosity of the prepared Pd/AC catalysts were determined using N_2_ adsorption–desorption experiments at −196 °C using an ASAP 2460 (Micromeritics, Norcross, GA, USA) surface area and porosity analyzer. Prior to each adsorption experiment, the samples were outgassed under vacuum at 150 °C overnight. The BET method was used to calculate the *S_BET_* values of the catalysts. The *V_total_* was accumulated up to a partial pressure of 0.95. The *D_p_* of the catalysts was analyzed using the non-linear density function theory method. The Pd particle size and size distribution of the prepared Pd/AC catalysts were determined using a TEM-2100Plus (JEOL, Tokyo, Japan) instrument with an accelerating voltage of 200 kV. The particle size distribution of the catalysts was obtained using the ImageJ (National Institutes of Health (NIH), Bethesda, MD, USA) software. The Pd dispersion was measured using the CO pulse chemisorption technique utilizing a Riken R6015 (Ohkura, Japan) instrument and assuming a CO/Pd stoichiometric ratio of 1:1. The prepared Pd/AC catalysts were reduced at 300 °C prior to analysis. The reduced catalysts were pulsed using a 10 vol.% CO/He flow until no more CO was adsorbed according to the TCD data. The acidity of the catalysts was measured via the NH_3_-TPD analysis using the BELCAT-B (BEL, Japan) chemisorption analyzer. The prepared Pd/AC catalysts (0.1 g) were treated at 300 °C under a He flow for 1 h. After cooling them to 100 °C, the samples were saturated with NH_3_ using a 10% NH_3_/He flow. The physically adsorbed or weakly bound NH_3_ was purged with He at 100 °C for 1 h. For the NH_3_-TPD measurements, the NH_3_ signals were monitored using both TCD and MS in the temperature range of 100–800 °C at a ramping rate of 5 °C min^−1^ under a 30 mL min^−1^ He flow. The chemical states of prepared Pd catalysts were examined using an AXIS Supra (Kratos, Kyoto, Japan) XPS instrument with monochromatic Al K_α_ (hν = 1486.6 eV) radiation. The XPS measurements were performed in the ultrahigh vacuum system (1 × 10^−^^7^ Pa) equipped with a hemispherical analyzer. The samples were mounted on a carbon tape, followed by degassing under vacuum and transferring to the analysis chamber for the XPS measurement. The energy resolution of XPS instrument was examined using Ag 3d_5/2_. The result showed that the full width at half maximum (FWHM) of the Ag 3d_5/2_ band was 0.96 eV. The XPS spectra of Figure 5a,c were analyzed at an energy step of 1 eV and those of Figure 5b,d, and Figure S2 (Supplementary) were analyzed at an energy step of 0.1 eV. The BE was calibrated by the C 1s peak (284.6 eV). The spectra were analyzed and processed using the ESCApe (Shimadzu-Kratos, Kyoto, Japan) software. Background subtraction was performed according to Shirley algorithm. For the determination of the binding energies, the Pd 3d, Cl 2p, C 1s, and O 1s spectra were fitted using Gaussian–Lorentz.

### 3.4. Partial Hydrogenation of Palm-BDF

The catalytic performance of the prepared Pd/AC catalysts for the partial hydrogenation of palm-BDF was evaluated using a batch-type glass reactor at 80 °C and 0.5 MPa of H_2_ under vigorous stirring. The amounts of palm-BDF and reduced catalysts were maintained at 150 and 1 g, respectively, for all experiments. The FAME compositions of palm-BDF and H-FAME were analyzed using a 6890 N (Agilent Technologies Inc., Santa Clara, CA, USA) gas chromatography instrument, equipped with a flame ionization detector and a 100 m × 0.25 mm HP-88 (Agilent J&W column, Agilent Technologies Inc., Santa Clara, CA, USA) fused-silica capillary column. The injector and detector temperatures were set at 250 °C, and the split ratio was 50:1. A 1 µL sample was injected into an oven and was maintained at 155 °C for 20 min. Subsequently, the temperature was increased to 230 °C at a ramping rate of 2 °C min^−1^ and was maintained for 10 min. The FAME compositions of the palm-BDF and H-FAME samples were qualitatively analyzed by comparing the obtained results with those of reference compounds according to the EN 14,103 international standard method. The oxidative stability of palm-BDF and H-FAME was analyzed using a 743 Rancimat (Metrohm, Herisau, Switzerland) instrument utilizing the EN 14,112 international standard method. The cloud point is the temperature at which the first biowax crystal appears in BDFs, and is frequently used to evaluate the cold flow properties of fuels. In this study, we measured the cloud point of the palm-BDF and H-FAME samples using a CPP 5GS (ISL PAC, USA) automated cloud point and pour point analyzer utilizing the ASTM D2500 method.

## 4. Conclusions

AC-supported Pd catalysts were prepared and used for the partial hydrogenation of a commercial palm-BDF into H-FAME, which had low poly-FAME components and presented high oxidation stability and suitable cold flow properties. We determined that the Pd precursors affected the particle sizes and chemical states of Pd in the prepared Pd/AC catalysts. The 1%Pd/AC (nit) catalyst with large Pd particles (8.4 nm) obtained using Pd(NO_3_)_2_ as precursor presented high activity and selectivity toward mono-FAME. Therefore, 1%Pd/AC (nit) can be a good candidate for the large-scale production of H-FAME with low operation cost and facile recycling of the spent Pd catalyst. The opposite was observed for the 1%Pd/AC (amc) catalyst, which contained small Pd particles with little Cl residues and presented low activity and poor selectivity for the synthesis of H-FAME. We further clarified that the performance of prepared Pd/AC catalysts in the synthesis of H-FAME were mainly influenced by the Pd particle size, and large Pd particles (approximately 8 nm) gave high activity and selectivity for conversion of poly-FAME components to mono-FAME components, particularly *cis*-products, as the targeting components of H-FAME. Meanwhile, the differences in activity and product selectivity between the Cl-containing and Cl-free Pd catalysts were negligibly small.

## Figures and Tables

**Table 1:** Characteristics of activated carbon (AC) and Pd/AC catalysts.

Sample	Pd (wt.%)	Cl ^1^ (wt.%)	*S_BET_* (m^2^ g^−1^)	*D_p_* (nm)	*V_total_* (cm^3^ g^−1^)	Pd Dispersion ^2^ (%)	Pd Size (nm)		Acidity	Pd^0^/Pd^2+^ Ratio ^5^
							CO Chem. ^2^	TEM ^3^	(mmol g^−1^) ^4^	
AC	-	n.d.	1782	0.87	0.70	**-**	-	-	0.01	-
1%Pd/AC (nit)	0.92	n.d.	1746	0.80	0.63	5.8	8.3	8.4 ± 1.5	0.10	3.6
1%Pd/AC (amc)	1.03	0.11	1777	0.80	0.70	59	0.8	2.7 ± 0.8	0.12	2.8
5%Pd/AC (com)	4.82	n.d.	919	0.77	0.46	15	3.3	-	0.51	-

^1^ Not detectable (n.d.). The Cl content was lower than the detection limit of the X-ray fluorescence instrument (<0.05 wt.%). ^2^ Measured using the CO pulse chemisorption method. ^3^ Determined using transmission electron microscopy (TEM). ^4^ Determined using NH_3_ temperature-programmed desorption (NH_3_-TPD). ^5^ Determined using XPS.

**Table 2:** Kinetic data and *S* values of the prepared Pd/AC catalysts ^1^.

	1%Pd/AC (nit)	1%Pd/AC (amc)	5%Pd/AC (com)
Initial rate (mmol g_pd_^−1^ s^−1^) ^2^	544	258	307
Turnover frequency (TOF) (s^−1^) ^2^	88	4.1	19
Ratio of *cis*-/*trans*-C_18:1_^3^	1.4	1.3	n.d. ^4^
Degree of complete hydrogenation (%) ^3^	16.1	157	n.d. ^4^
*k_1_* (s^−1^ g^−1^)	0.23	0.082	0.064
*k_2_* (s^−1^ g^−1^)	0.091	0.136	n.d. ^4^
*S = k*_1_ */k*_2_	2.6	0.60	n.d. ^4^

^1^ The reaction conditions were: 80 °C, 0.5 MPa of H_2_, 1 g of catalyst, and 150 g of oil. ^2^ Calculated at the first 5 min of reaction, when the poly-FAME conversions were lower than 30%. ^3^ Calculated at the poly-FAME conversion of 90% at the reaction times of 16 and 26 min for 1%Pd/AC (nit) and 1%Pd/AC (amc), respectively. ^4^ These data were not detectable (n.d.) because the poly-FAME conversion over a reference catalyst of 5%Pd/AC (com) did not reach 90% at the reaction time of 120 min.

**Table 3:** FAME composition and fuel properties of palm-biodiesel fuel (BDF) and H-FAME over prepared Pd/AC catalysts at a poly-FAME conversion of 90%.

	Palm-BDF	H-FAME			
		1%Pd/AC (amc) ^1^	1%Pd/AC (nit) ^1^	2%Pd/CA ^2^	2%Pd/AC ^2^
Pd size (nm)	-	2.7	8.4	10	18
FAME composition (wt.%)					
C_18:3_	0.1	0.0	0.0	-	-
C_18:2_	8.5	0.9	0.9	-	-
Total C_18:1_	36.2	17.1	37.8	-	-
*cis*-C_18:1_	36.1	9.5	22.0	-	-
*trans*-C_18:1_	0.1	7.6	15.8	-	-
C_18:0_	4.8	31.6	10.9	-	-
Poly-FAME	8.6	0.9	0.9	0	0
Mono-FAME	36.2	17.1	38.0	4.26	28.1
Sat-FAME	52.6	81.4	60.9	95.7	71.9
Fuel properties					
Oxidative stability (h)	13	61	60	41	36
Cloud point (°C)	15	28	18	26	23

^1^ The reaction conditions were: 80 °C, 0.5 MPa of H_2_, 1 g of catalyst, and 150 g of oil (corresponding to a catalyst weight of 0.67 wt.% in the reaction).^2^ The reaction conditions were: 120 °C, 0.4 MPa of H_2_, 1.5 g of catalyst, and 100 g of oil (corresponding to a catalyst weight of 1.5 wt.% in the reaction) [34]. The 2%Pd/AC catalyst represented the activated carbon (AC)-supported Pd catalyst with a Pd loading of 2 wt.%. The 2%Pd/CA catalyst represented the carbon aerogel (CA)-supported Pd catalyst with a Pd loading of 2 wt.%.

## Data Availability

Not applicable.

## Supplementary Materials

Supplementary Materials can be found at https://www.mdpi.com/1422-0067/22/3/1256/s1.
