# Supplementary material for: Effect of Pd Precursor Salts on the Chemical State, Particle Size, and Performance of Activated Carbon-Supported Pd Catalysts for the Selective Hydrogenation of Palm Biodiesel
Source: Int J Mol Sci. 2021 Jan 27;22(3):1256. doi: 10.3390/ijms22031256 (PMC7865769; doi:10.3390/ijms22031256)
Supplement: Supplementary file 1 [file ijms-22-01256-s001.pdf]

Supplementary Information

Effect of Pd precursor salts on the chemical state, particle size, and performance of activated carbon-supported Pd catalysts for the selective hydrogenation of palm biodiesel

Parncheewa Udomsap, Apiluck Eiad-Ua,* Shih-Yuan Chen,* Takehisa Mochizuki,* Nuwong Chollacoop, Yuji Yoshimura, Masayasu Nishi, Hiroyuki Tateno and Hideyuki Takagi

Table of Contents

Preparation of 1%Pd/AC (nit) and 1%Pd/AC (amc) catalysts……………………….2

Preparation of Cl-containing 1%Pd/AC (nit) and Cl-free 1%Pd/AC (amc)………..2

Table S1. The elemental analysis of 1%Pd/AC(nit) and 1%Pd/AC(amc) catalysts and corresponding Cl-containing and Cl-free counterparts…………………………………..3

Fig. S1. Temperature dependence on the formation of CH_4, CO and CO_2 over the 1%Pd/AC(nit) catalyst during the H_2-TPR experiment. The CH_4, CO and CO_2 were monitored by mass spectrometer using m/z ratios of 16, 28 and 44, respectively………4

Fig. S2. XPS analysis of the chemical states of Cl in 1%Pd/AC (amc). The dark gray and light gray areas under the fitting curves illustrate the Cl–Pd and Cl–organic compound bonds……………………………………………………………………………………..5

Fig. S3. XRD patterns of used Pd/AC (nit) and Pd/AC (amc) catalysts…………………..6

Fig. S4. (a) Poly-FAME conversion as a function of reaction time, and percentages of (b) mono-FAME (c) *cis*-mono-FAME and (d) sat-FAME as functions of the poly-FAME conversion over the Cl-containing catalysts of (□) 1%Pd/AC (nit)-Cl and (●) 1%Pd/AC (amc), and the Cl-free catalysts of (■) 1%Pd/AC (nit) and (○) 1%Pd/AC (amc)-noCl…...7

Preparation of 1%Pd/AC (nit) and 1%Pd/AC (amc) catalysts

The preparation of 1%Pd/AC(nit) and 1%Pd/AC(amc) catalysts was used the same manner of section 3.2, which is described below. Before impregnation, AC was dried at 110 °C. Two Pd/AC catalysts with Pd loadings of 1 wt.% were prepared via the impregnation of Pd aqueous solutions on AC under vacuum (approximately 0.1 torr) at 25 °C followed by aging for 24 h. The Pd aqueous solutions were obtained by dissolving 0.022 g of $Pd(NO_3)_2{\cdot}xH_2O$ and 0.025 g $Pd(NH_3)_4Cl_2{\cdot}xH_2O$ precursors in 0.4 mL of deionized water. The samples were subsequently dried at 60 °C for 6 h using a rotary evaporator followed by calcination at 300 °C in a N_2 flow for 2 h. The prepared samples were labeled 1%Pd/AC (nit) and 1%Pd/AC (amc), where (nit) and (amc) indicate that $Pd(NO_3)_2{\cdot}xH_2O$ and $Pd(NH_3)_4Cl_2{\cdot}xH_2O$ were used as precursors, respectively. Prior to using the catalysts for the partial hydrogenation of palm-FAME, they were reduced at 300 °C for 1 h under a H_2 flow of 50 mL min^{-1}.

Preparation of Cl-containing 1%Pd/AC (nit) and Cl-free 1%Pd/AC (amc)

For deep understanding of the effect of Cl residue, the as-prepared 1%Pd/AC (nit) catalyst was impregnated by an aqueous solution (NH_4Cl), and the loading of NH_4Cl was 0.1 wt.%. Then, the sample was dried at 110 °C for 4 h, followed by reduced at 300 °C for 1 h under a H_2 flow of 50 mL min^{-1}. As a result, the Cl-containing 1%Pd/AC (nit) catalyst (termed 1%Pd/AC (nit)-Cl)) was obtained. The XPS analysis indicated that the 1%Pd/AC (nit)-Cl catalyst contains approximately 0.08 wt.% of Cl.

On the other hand, the as-prepared 1%Pd/AC (amc) catalyst was further washed by 0.1 M of NH_4OH solution to remove the Cl residues. The washed sample was dried at 110 °C for 4 h, followed by reduced at 300 °C for 1 h under a H_2 flow of 50 mL min^{-1}. As a result, the Cl-free 1%Pd/AC (amc) catalyst (termed 1%Pd/AC (amc)-noCl) was obtained. The XPS analysis indicated that the Cl concentration of 1%Pd/AC (amc)-noCl catalyst was lower than the detection limit.

Table S1. The elemental analysis of 1%Pd/AC(nit) and 1%Pd/AC(amc) catalysts and corresponding Cl-containing and Cl-free counterparts.

Sample	Pd (wt.%)	Cl[1] (wt.%)
AC	-	n.d. [2]
1%Pd/AC (nit)	0.92	n.d. [2]
1%Pd/AC (nit)-Cl	0.87	0.08
1%Pd/AC (amc)	1.03	0.11
1%Pd/AC (amc)-noCl	0.95	n.d. [2]

[1] Determined by the XPS analysis.

[2] Not detectable (n.d.). The Cl content was lower than the detection limit of the X-ray fluorescence instrument (< 0.05 wt.%).

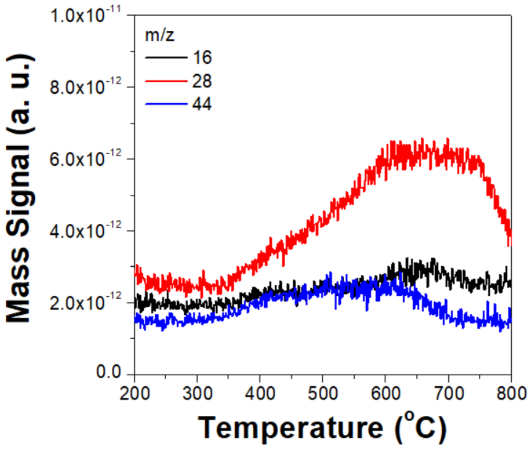

Fig. S1. Temperature dependence on the formation of CH_4, CO, and CO_2 over the 1%Pd/AC(nit) catalyst during the H_2-TPR experiment. The CH_4, CO, and CO_2 were monitored by mass spectrometer using m/z ratios of 16, 28, and 44, respectively.

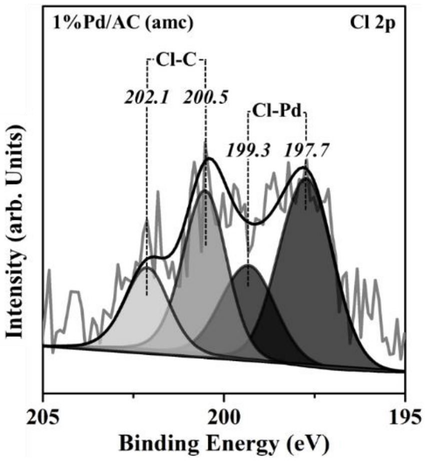

Fig. S2. XPS analysis of the chemical states of Cl in 1%Pd/AC (amc). The dark gray and light gray areas under the fitting curves illustrate the Cl–Pd and Cl–organic compound bonds.

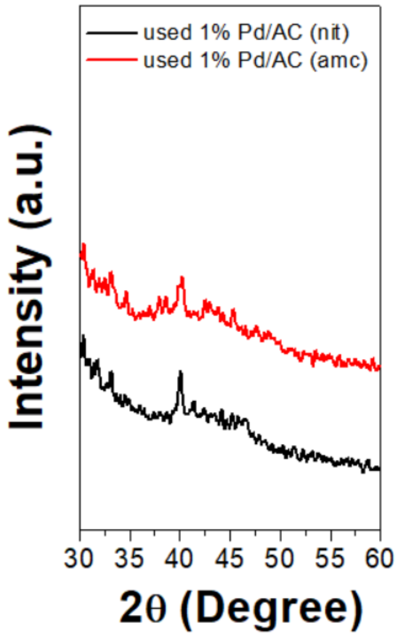

Fig. S3. XRD patterns of used Pd/AC (nit) and Pd/AC (amc) catalysts.

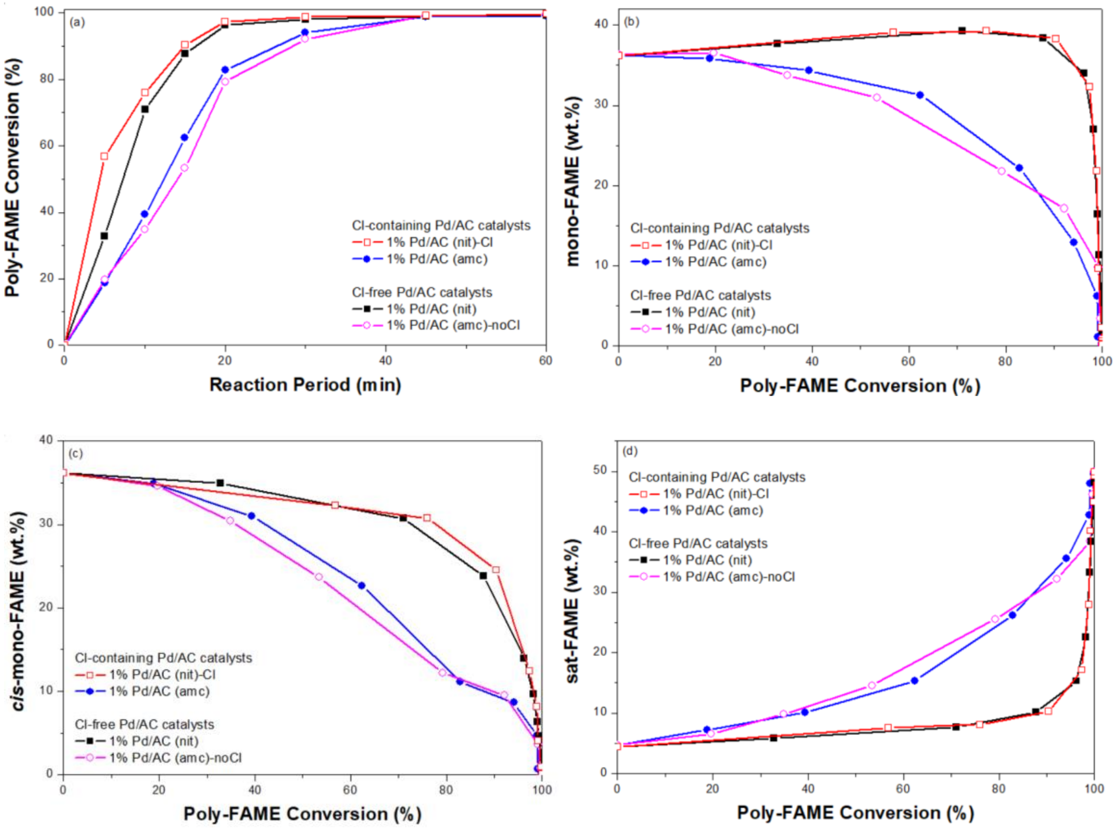

Fig. S4. (a) Poly-FAME conversion as a function of reaction time, and percentages of (b) mono-FAME, (c) *cis*-mono-FAME, and (d) sat-FAME as functions of the poly-FAME conversion over the Cl-containing catalysts of (□) 1%Pd/AC (nit)-Cl and (●) 1%Pd/AC (amc), and the Cl-free catalysts of (■) 1%Pd/AC (nit) and (○) 1%Pd/AC (amc)-noCl.
